# Supplementary material for: Causal Relationship between Aging and Anorexia Nervosa: A White-Matter-Microstructure-Mediated Mendelian Randomization Analysis
Source: Biomedicines. 2024 Aug 16;12(8):1874. doi: 10.3390/biomedicines12081874 (PMC11351342; doi:10.3390/biomedicines12081874)
Supplement: Supplementary file 1 [file biomedicines-12-01874-s001.zip › Supplementary Figure/Figure S4/Singe SNP plot of MR analysis for TL to ubm-b-1911.pdf]

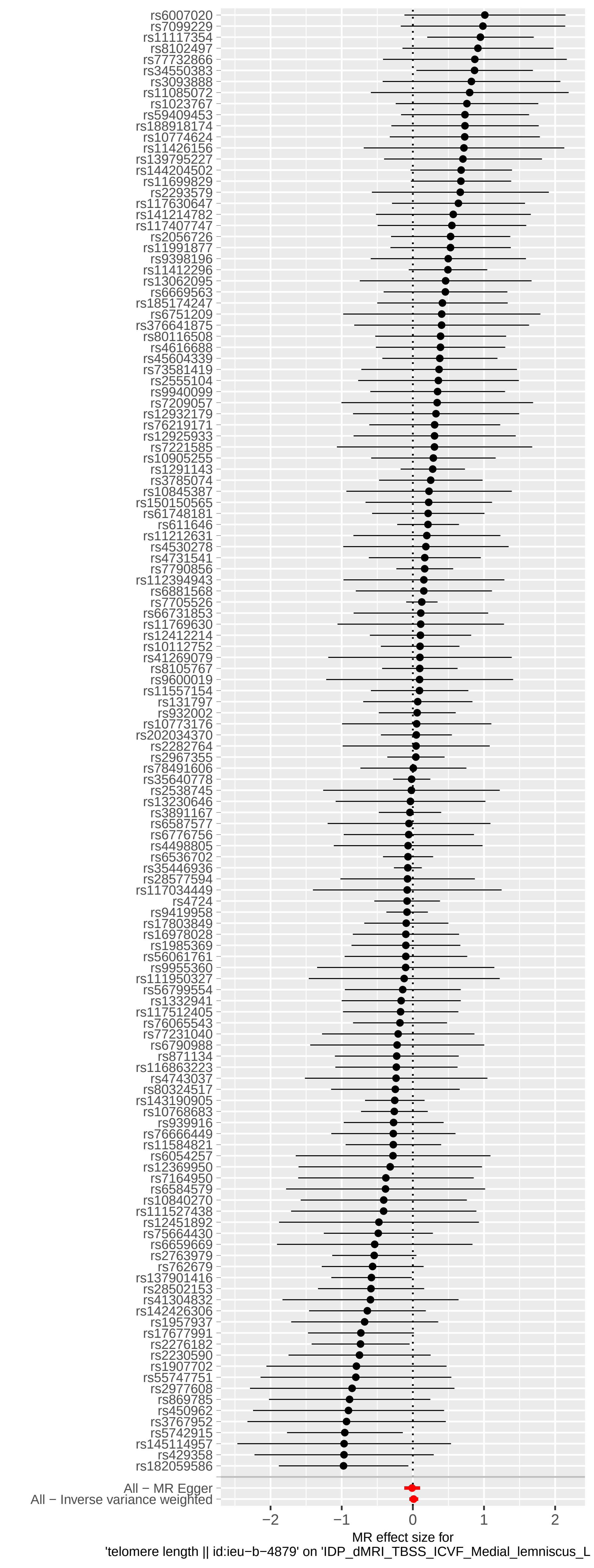
rs6007020
rs7099229
rs11117354
rs8102497
rs77732866
rs34550383
rs3093888
rs11085072
rs1023767
rs59409453
rs188918174
rs10774624
rs11426156
rs139795227
rs144204502
rs11699829
rs2293579
rs117630647
rs141214782
rs117407747
rs2056726
rs11991877
rs9398196
rs11412296
rs13062095
rs6669563
rs185174247
rs6751209
rs376641875
rs80116508
rs4616688
rs45604339
rs73581419
rs2555104
rs9940099
rs7209057
rs12932179
rs76219171
rs12925933
rs7221585
rs10905255
rs1291143
rs3785074
rs10845387
rs150150565
rs61748181
rs611646
rs11212631
rs4530278
rs4731541
rs7790856
rs112394943
rs6881568
rs7705526
rs66731853
rs11769630
rs12412214
rs10112752
rs41269079
rs8105767
rs9600019
rs11557154
rs131797
rs932002
rs10773176
rs202034370
rs2282764
rs2967355
rs78491606
rs35640778
rs2538745
rs13230646
rs3891167
rs6587577
rs6776756
rs4498805
rs6536702
rs35446936
rs28577594
rs117034449
rs4724
rs9419958
rs17803849
rs16978028
rs1985369
rs56061761
rs9955360
rs111950327
rs56799554
rs1332941
rs117512405
rs76065543
rs77231040
rs6790988
rs871134
rs116863223
rs4743037
rs80324517
rs143190905
rs10768683
rs939916
rs76666449
rs11584821
rs6054257
rs12369950
rs7164950
rs6584579
rs10840270
rs111527438
rs12451892
rs75664430
rs6659669
rs2763979
rs762679
rs137901416
rs28502153
rs41304832
rs142426306
rs1957937
rs17677991
rs2276182
rs2230590
rs1907702
rs55747751
rs2977608
rs869785
rs450962
rs3767952
rs5742915
rs145114957
rs429358
rs182059586
All − MR Egger
All − Inverse variance weighted
−2
−1
0
1
2
MR effect size for
'telomere length || id:ieu−b−4879' on 'IDP_dMRI_TBSS_ICVF_Medial_lemniscus_L
